# Respiratory Syncytial Virus-Induced Oxidative Stress Leads to an Increase in Labile Zinc Pools in Lung Epithelial Cells

**DOI:** 10.1128/mSphere.00447-20

**Published:** 2020-05-27

**Authors:** Naseem Ahmed Khan, Mohit Singla, Sweety Samal, Rakesh Lodha, Guruprasad R. Medigeshi

**Affiliations:** a Clinical and Cellular Virology Laboratory, Translational Health Science and Technology Institute, Faridabad, Haryana, India; b Department of Pediatrics, All India Institute of Medical Sciences, New Delhi, India; c Infection and Immunity Division, Translational Health Science and Technology Institute, Faridabad, Haryana, India; University of Michigan—Ann Arbor

**Keywords:** ROS, TPEN, ZIP, labile zinc, respiratory syncytial virus

## Abstract

Zinc supplementation in cell culture has been shown to inhibit various viruses, like herpes simplex virus, rotavirus, severe acute respiratory syndrome (SARS) coronavirus, rhinovirus, and respiratory syncytial virus (RSV). However, whether zinc plays a direct antiviral role in viral infections and whether viruses have adopted strategies to modulate zinc homeostasis have not been investigated. Results from clinical trials of zinc supplementation in infections indicate that zinc supplementation may be beneficial in a pathogen- or disease-specific manner, further underscoring the importance of understanding the interaction between zinc homeostasis and virus infections at the molecular level. We investigated the effect of RSV infection on zinc homeostasis and show that RSV infection in lung epithelial cells leads to modulation of zinc homeostasis. The intracellular labile zinc pool increases upon RSV infection in a multiplicity of infection (MOI)-dependent fashion. Small interfering RNA (siRNA)-mediated knockdown of the ubiquitous zinc uptake transporter *ZIP1* suggests that labile zinc levels are increased due to the increased uptake by RSV-infected cells as an antiviral response. Adding zinc to culture medium after RSV infection led to significant inhibition of RSV titers, whereas depletion of zinc by a zinc chelator, *N*,*N*,*N*′,*N*′-tetrakis(2-pyridinylmethyl)-1,2-ethanediamine (TPEN) led to an increase in RSV titers. The inhibitory effect of zinc was specific, as other divalent cations had no effect on RSV titers. Both RSV infection and zinc chelation by TPEN led to reactive oxygen species (ROS) induction, whereas addition of zinc blocked ROS induction. These results suggest a molecular link between RSV infection, zinc homeostasis, and oxidative-stress pathways and provide new insights for developing strategies to counter RSV infection.

**IMPORTANCE** Zinc deficiency rates in developing countries range from 20 to 30%, and zinc supplementation trials have been shown to correct clinical manifestations attributed to zinc deficiency, but the outcomes in the case of respiratory infections have been inconsistent. We aimed at understanding the role of zinc homeostasis in respiratory syncytial virus (RSV) infection. Infection of lung epithelial cell lines or primary small-airway epithelial cells led to an increase in labile zinc pools, which was due to increased uptake of zinc. Zinc supplementation inhibited RSV replication, whereas zinc chelation had an opposing effect, leading to increases in RSV titers. Increases in labile zinc in RSV-infected cells coincided with induction of reactive oxygen species (ROS). Both zinc depletion and addition of exogenous ROS led to enhanced RSV infection, whereas addition of the antioxidant inhibited RSV, suggesting that zinc is part of an interplay between RSV-induced oxidative stress and the host response to maintain redox balance.

## Abstract (precis)

Zinc deficiency rates in developing countries range from 20 to 30%, and zinc supplementation trials have been shown to correct clinical manifestations attributed to zinc deficiency, but the outcomes in the case of respiratory infections have been inconsistent. We aimed at understanding the role of zinc homeostasis in respiratory syncytial virus (RSV) infection. Infection of lung epithelial cell lines or primary small-airway epithelial cells led to an increase in labile zinc pools, which was due to increased uptake of zinc. Zinc supplementation inhibited RSV replication, whereas zinc chelation had an opposing effect, leading to increases in RSV titers. Increases in labile zinc in RSV-infected cells coincided with induction of reactive oxygen species (ROS). Both zinc depletion and addition of exogenous ROS led to enhanced RSV infection, whereas addition of the antioxidant inhibited RSV, suggesting that zinc is part of an interplay between RSV-induced oxidative stress and the host response to maintain redox balance.

## INTRODUCTION

Respiratory syncytial virus (RSV) is a common cause of acute lower respiratory tract infection (ALRI) in infants, which leads to hospitalization. Elderly and immunocompromised individuals are also highly susceptible to severe disease due to RSV. According to estimates for the year 2015, RSV-ALRI led to 1.4 million hospital admissions of children less than 6 months of age, of which 27,000 succumbed to infection (1). RSV belongs to the *Paramyxoviridae* family and is an enveloped, nonsegmented, negative-strand RNA virus. The clinical manifestations of RSV infection vary from mild upper respiratory tract illness (URTI) to potentially life-threatening lower respiratory tract involvement (LRTI). There is no vaccine or effective antiviral drug available for RSV; the only available treatment is immunoprophylaxis of severe RSV disease in high-risk infants with palivizumab (2, 3), which is not an affordable option in many low- and middle-income countries. Therefore, there is a need to develop affordable interventions through better understanding of cellular factors that regulate RSV infection.

Zinc is an essential micronutrient and plays diverse physiological roles in multiple cellular processes, such as the immune response, signal transduction, organelle homeostasis, cell proliferation, and cell death (4, 5). Zinc deficiency rates in developing countries range from 20 to 30%. In India, studies have reported that 50 to 75% of pregnant women and that between 40 and 75% of children are zinc deficient (6). Nearly 30% of healthy elderly subjects may be zinc deficient in developed countries. As per the World Health Organization estimates, 800,000 people die annually due to zinc deficiency, and more than half of these deaths occur in children under the age of 5 years (7). Zinc supplementation was shown to reduce the respiratory morbidity of ALRI in children less than 5 years of age who were zinc deficient (8). Studies examining the clinical effects of zinc for treating pneumonia in children have shown conﬂicting results, with some studies showing a beneﬁcial effect on the duration of recovery and severity but with other studies suggesting that zinc has no treatment beneﬁt (9–13). Although the necessary role of zinc as a micronutrient in various physiological functions has been demonstrated, the molecular mechanism underlying the effects of zinc during viral infections has not been elucidated. In this study, we utilized changes in intracellular labile zinc pools as a measure of zinc homeostasis in lung epithelial cell lines and primary small-airway epithelial cells (SAECs) and investigated the effect of RSV infection on zinc homeostasis. Our results suggest that zinc homeostasis plays a critical role in the host response to RSV infection by regulating oxidative stress and inhibiting virus replication.

## RESULTS

### Labile zinc pool increases in RSV infection in A549 cells.

**FIG 1:**
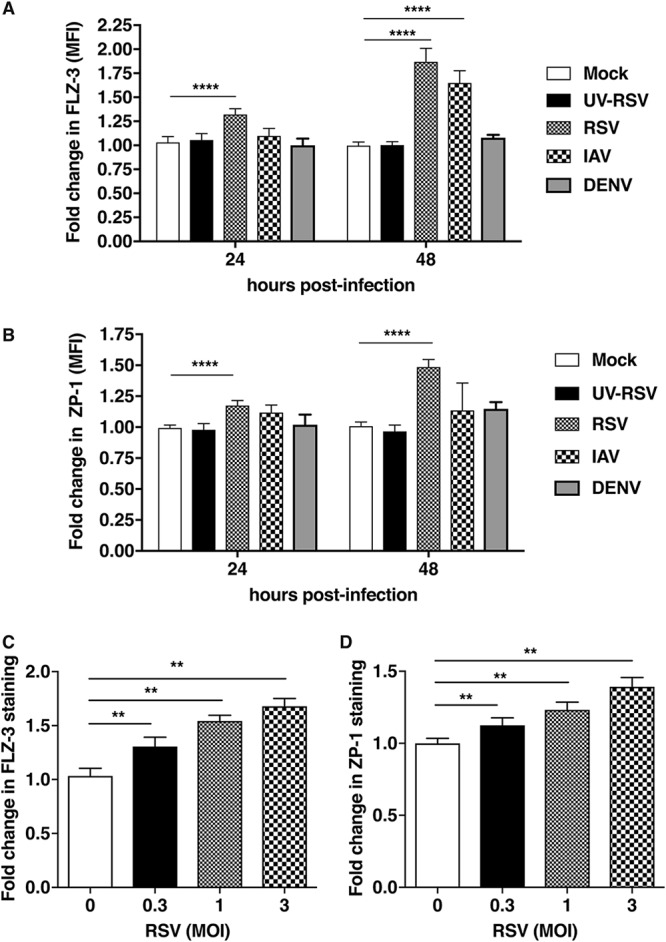
RSV infection leads to increases in labile zinc in A549 cells. A549 cells were infected with UV-inactivated RSV (UV-RSV) or wild-type RSV at an MOI of 0.3, with IAV at an MOI of 1, or with DENV at an MOI of 5, as indicated. Labile zinc levels were measured at 24 h p.i. and 48 h p.i. using FLZ-3 (A) and ZP-1 (B) by flow cytometry. A549 cells were infected with RSV at MOIs of 0.3, 1, and 3. Labile zinc levels were determined at 24 h p.i. by staining cells with FLZ-3 (C) and ZP-1 (D) by flow cytometry. Data are from at least two independent experiments. Error bars represent means with standard deviations. **, *P* < 0.01; ****, *P* < 0.0001.

There are contrasting reports on the role of zinc in viral infections, and we speculated that viral infections, especially those from RNA viruses, which replicate in the cytoplasm, may alter the dynamics of cytosolic levels of free zinc. A549 cells were infected with RSV at a multiplicity of infection (MOI) of 0.3, and under these conditions, we observed a time-dependent increase in the percentage of infected cells from 30% to 90% from 24 h to 48 h postinfection (p.i.) (see Fig. S1A in the supplemental material). We utilized two different zinc-binding fluorophores, fluozin-3 (FLZ-3) and zinpyr-1 (ZP-1), and measured labile zinc levels by flow cytometry in RSV-infected cells. We observed an increase in labile zinc levels in both FLZ-3 and ZP-1 stains in a time-dependent manner. Levels of FLZ-3 in stains increased from 30% to 70% from 24 h p.i. to 48 h p.i. (Fig. 1A) in RSV-infected cells. Similarly, ZP-1 staining showed an increase from 20% to 50% from 24 h p.i. to 48 h p.i. (Fig. 1B). To further confirm whether an increase in labile zinc levels is due to active RSV replication, we used UV-inactivated RSV, which failed to show any modulation in free zinc levels (Fig. 1A and B). We next confirmed whether this modulation of free zinc levels is specific to RSV by infecting A549 cells with influenza A virus subtype H1N1 (IAV) at an MOI of 1. Labile zinc levels were measured using FLZ-3 and ZP-1 at 24 h p.i. and 48 h p.i. IAV infection led to a significant increase in FLZ-3 staining only at 48 h p.i. (Fig. 1A), whereas ZP-1 staining did not show any significant change (Fig. 1B). We next tested whether the effect is observed with other nonrespiratory RNA viruses, such as dengue virus (DENV) at an MOI of 5, and measured free zinc levels as described above. We did not observe any change in labile zinc levels in cells infected with DENV (Fig. 1A and B), suggesting that zinc homeostasis is altered specifically by RSV and IAV; RSV infection in particular led to increases in both cytosolic and vesicular labile zinc pools, whereas DENV infection had no effect in A549 cells. Cell viability was not compromised under any of these conditions, as verified by staining cells with live-dead stain and examining them by flow cytometry (Fig. S1B). We next infected A549 cells with RSV at MOIs of 0.3, 1, and 3, and labile zinc levels were measured at 24 h p.i. using FLZ-3 and ZP-1. We observed an MOI-dependent increase in labile zinc levels. FLZ-3 staining increased from 30% to 50% at MOIs from 0.3 to 1, and a 70% increase in staining was observed at an MOI of 3 (Fig. 1C). Similarly, ZP-1 staining increased from 15% at an MOI of 0.3, to 25% at an MOI of 1, and to 40% at an MOI of 3 (Fig. 1D). We determined the percentages of cells infected under these conditions by immunofluorescence (IF) staining for RSV F protein. As expected, the proportion of RSV-infected cells increased from 40% at an MOI of 0.3 to 70% at an MOI of 1, and 95% of the cells were infected at an MOI of 3 (Fig. S1C). These results suggest that RSV infection triggers an increase in cellular free zinc levels that is time and MOI dependent; this increase in labile zinc levels is specific to RSV, as dengue infection had no effect.

10.1128/mSphere.00447-20.1 FIG S1 Percentages of RSV infection in A549 cells. (A) A549 cells were infected with UV-inactivated RSV or WT-RSV at an MOI of 0.3 or with DENV at an MOI of 5. Percentages of infection were determined by staining cells with RSV-F antibody for RSV and pan-flavivirus 4G2 antibody for DENV and then with secondary antibodies conjugated with Alexa Fluor-568 (red) at 24 h p.i. and 48 h p.i. Nuclei are stained with DAPI (4′,6-diamidino-2-phenylindole; blue). Scale bar, 100 μm. (B) A549 cells infected as described above were stained with eFluor780 dye, and cell viability was determined at 24 h p.i. and 48 h p.i. by flow cytometry. (C) A549 cells were infected with RSV at MOIs of 0.3, 1, and 3. Percentages of infection were determined at 24 h p.i. by staining as described above. Data are from at least two independent experiments. Error bars represent standard deviations from the means. Download FIG S1, JPG file, 2.2 MB. Copyright © 2020 Khan et al. 2020 Khan et al. This content is distributed under the terms of the Creative Commons Attribution 4.0 International license.

### Increases in free zinc levels in primary cells and nasal epithelial cells from RSV patients.

**FIG 2:**
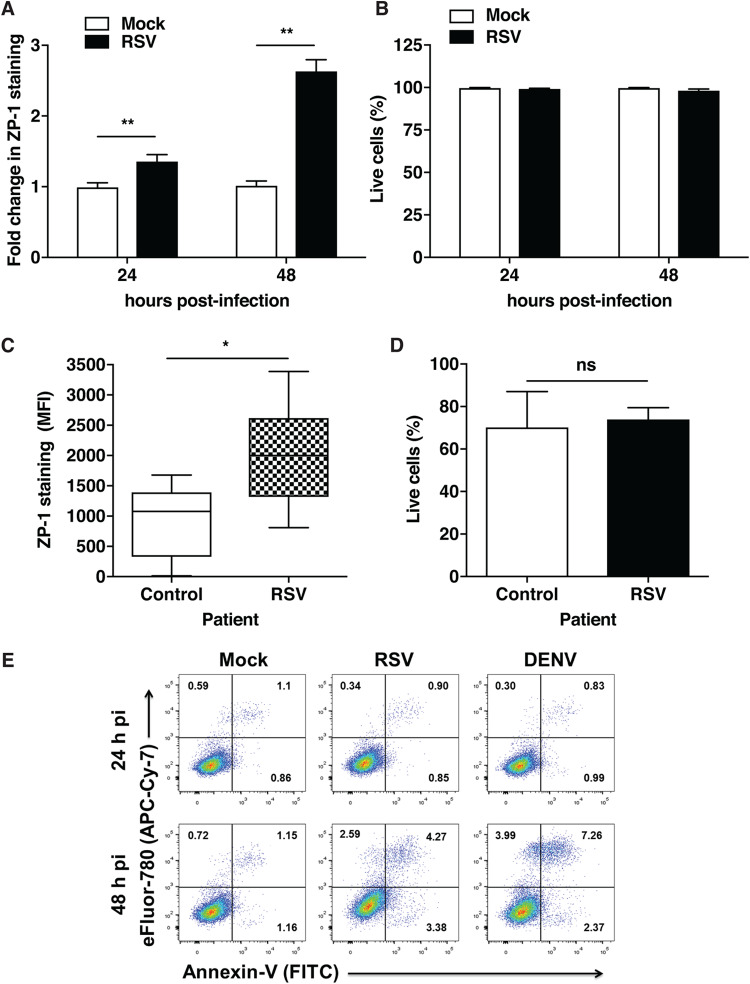
Labile zinc levels increase in primary epithelial cells. (A) SAECs were infected with RSV at an MOI of 3, and cells were stained with ZP-1 at 24 h p.i. and 48 h p.i. (B) Cell viability was determined by staining the cells with the fixable viability dye eFluor780. (C) Nasal epithelial cells isolated from nasopharyngeal aspirates were stained with ZP-1. Labile zinc levels are presented as median fluorescence intensities (MFI). (D) The cell viability of NPA specimens was determined by staining the cells with the fixable viability dye eFluor780. There were 10 controls and 7 cases. (E) A549 cells were infected with RSV and DENV at MOIs of 0.3 and 5, respectively. Cells were stained with Annexin-V and eFluor780 dye at 24 h p.i. and 48 h p.i. Cell populations represent different phases: healthy, early apoptosis, late apoptosis, and necrosis. Annexin-V signal was detected in FITC filter and eFluor-780 signal was detected in APC-Cy-7 filter. Data are from at least two independent experiments. *, *P* < 0.05; **, *P* < 0.01; ns, differences were not significant.

We observed increases in labile zinc levels in A549 cells infected with RSV. To further verify whether this phenomenon is specific to A549 cells, we used primary small-airway epithelial cells (SAECs) to further confirm these observations. SAECs were infected with RSV at an MOI of 3, and cells were stained with ZP-1 at 24 and 48 h p.i. Under these conditions, around 60% of the cells were infected with RSV (Fig. S2). As with A549 cells, we observed a time-dependent increase in labile zinc levels in SAECs infected with RSV without any effect on cell viability (Fig. 2A and B). We next isolated nasal epithelial cells from nasopharyngeal washes collected from pediatric patients (Table 1) infected with RSV or healthy controls. Labile zinc levels were measured by ZP-1 staining, and we observed a 2-fold increase in the ZP-1 signal in nasal epithelial cells isolated from RSV patients compared to levels in cells from healthy controls (Fig. 2C), and as observed for A549 cells and SAECs, the viability of nasal epithelial cells that were used for ZP-1 staining was not compromised under the experimental conditions (Fig. 2D). Overall, these data suggest that RSV infection leads to an increase in labile zinc levels in cell lines, primary cells, and nasal epithelial cells isolated from RSV patients.

**TABLE 1:** Clinical characteristics of patients with RSV infection

Parameter	Value for:	
	Controls	Cases
No. of patients	10	7
Median age (mo) (IQR)	9 (3.8–15.8)	3 (2–15)
Male/female ratio	9:1	7:0

10.1128/mSphere.00447-20.2 FIG S2 Percentages of RSV infection in SAECs. SAECs were infected with RSV at an MOI of 3. Percentages of infection were determined using immunofluorescence. Cells were stained with RSV-F antibody followed by secondary antibodies conjugated with Alexa Fluor-488 (green) at 24 h p.i. Nuclei are stained with DAPI (blue). Scale bar, 100 μm. Download FIG S2, TIF file, 1.5 MB. Copyright © 2020 Khan et al. 2020 Khan et al. This content is distributed under the terms of the Creative Commons Attribution 4.0 International license.

Apoptosis has been shown to induce an increase in labile zinc levels (14, 15). Although cell viability was not affected under our experimental conditions, we further verified whether increases in labile zinc pools are due to apoptosis induction by RSV. We infected A549 cells with RSV and DENV at MOIs of 0.3 and 5, respectively, and stained cells with Annexin-V to measure the proportions of apoptotic cells under these conditions. As observed earlier, there was no significant difference in the nonviable cell populations between RSV and DENV (2.59% versus 3.99%). The proportion of Annexin-V-positive cells increased from 1.15% to 4.27% in cells infected with RSV and to 7.26% in cells infected with DENV, clearly indicating that induction of apoptosis does not contribute to induction of labile zinc levels in RSV infections (Fig. 2E). As a positive control for this assay, we induced apoptosis in A549 cells by heat stress as per previous studies (16, 17), which led to Annexin-V positivity in 83% of the cells (Fig. S3A). Under these conditions, cells stained with FLZ-3 showed a 4-fold increase in staining, whereas cells stained with ZP-1 showed an ∼12-fold increase in signal intensity (Fig. S3B). These results suggest that although apoptosis leads to increases in labile zinc levels, it does not contribute to increases in labile zinc levels observed in RSV infection.

10.1128/mSphere.00447-20.3 FIG S3 Effect of apoptosis on labile zinc pools. (A) A549 cells were incubated at 55^○^C for 15 min. Cells were stained with Annexin-V and eFluor-780 dye. (B) Heat-treated cells were stained with FLZ-3 or ZP-1 along with eFluor-780. Fold changes in labile zinc levels were calculated in live-cell populations. Data are from two independent experiments. Error bars represent standard deviations from the means. **, *P* < 0.01. Download FIG S3, TIF file, 1.3 MB. Copyright © 2020 Khan et al. 2020 Khan et al. This content is distributed under the terms of the Creative Commons Attribution 4.0 International license.

### *ZIP1* KD upregulates RSV infection.

**FIG 3:**
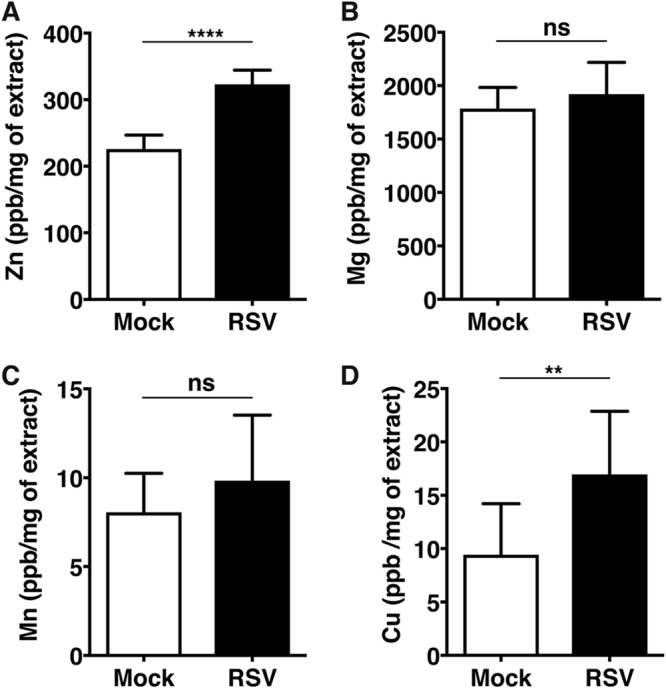
Total zinc content increases in RSV infection. (A to D) A549 cells were infected with RSV at an MOI of 0.3. The total content of the indicated divalent cations was determined by ICP-MS in RSV-infected A549 cells at 48 h p.i. Data are from at least two independent experiments. Error bars represent standard deviations of the means. **, *P* < 0.01; ****, *P* < 0.0001.

**FIG 4:**
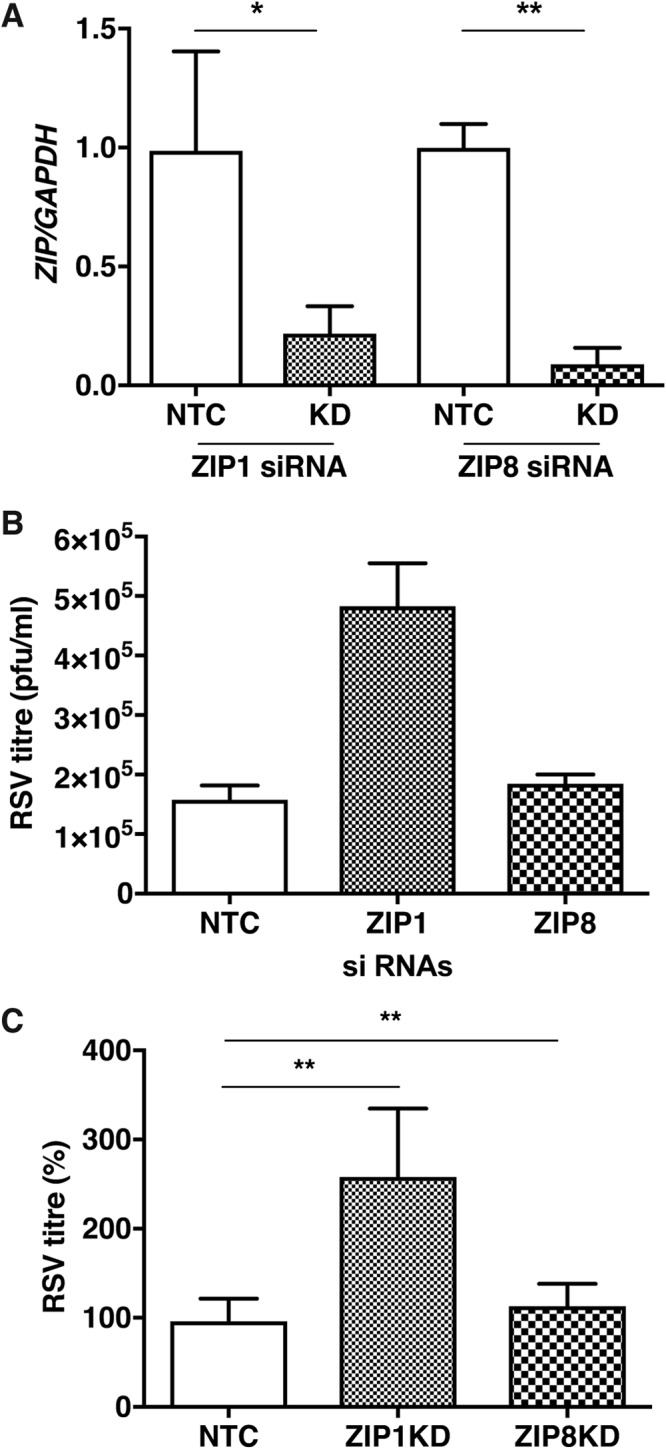
Knockdown of *ZIP1* inhibits RSV infection in A549 cells. (A) A549 cells were treated with siRNA specific to *ZIP1*, *ZIP8*, and scrambled siRNA as a control (labeled as NTC [nontargeted control]). *ZIP1* and *ZIP8* knockdown (KD) was confirmed at the mRNA level by qRT-PCR. (B) A549 cells were treated with *ZIP1* and *ZIP8* siRNA and later infected with RSV at an MOI of 0.3. A representative viral titer is represented in numbers of PFU per milliliter. (C) RSV titers from two independent experiments from *ZIP1* and *ZIP8* knockdown conditions. Error bars represent standard deviations of the means. *, *P* < 0.05; **, *P* < 0.01.

We next evaluated whether the increase in labile zinc pools observed in RSV infection is due to enhanced zinc uptake. To test this, we utilized inductively coupled plasma mass spectrometry (ICP-MS) to quantify total zinc content within the cell under RSV infection conditions. A549 cells were infected with RSV and DENV at MOIs of 0.3 and 5, respectively. Total metal content was determined at 48 h p.i. The amount of Zn, Mg, Mn, and Cu detected by ICP-MS in parts per billion was normalized to the total protein content of the cells to account for any difference in cell numbers. We observed an increase in total zinc content from 224 ± 22 ppb/mg (mean ± standard deviation [SD]) in mock-infected cells to 322 ± 22 ppb/mg in RSV-infected cells, but no significant difference was observed in Mg or Mn content between these cells. Surprisingly, we also observed a significant increase in Cu content in RSV infection relative to that in uninfected cells (9.4 ± 4.8 ppb/mg versus 16.9 ± 6 ppb/mg) (Fig. 3A to D). This suggests that an increase in the total zinc content in RSV-infected cells is possibly due to zinc uptake. Zinc homeostasis in cells is regulated by 14 influx (*SLC39* or *ZIP*) and 10 efflux (*SLC30* or *ZNT*) transporters and also by redistribution between intracellular organelles (18–20). Because the *ZIP* family of transporters plays a key role in zinc uptake, we next performed small interfering RNA (siRNA)-mediated knockdown (KD) of *ZIP1*, which is a ubiquitously expressed zinc uptake transporter localized to the plasma membrane, and of *ZIP8*, whose expression is higher in lungs than in other tissues and is also mostly localized to the plasma membrane. We infected *ZIP1* and *ZIP8* KD cells with RSV at an MOI of 0.3. RSV titers were measured at 24 h p.i. by plaque assays. siRNA transfections led to an approximately 80 to 90% knockdown efficiency in the respective *ZIP1* and *ZIP8* mRNAs compared to that of nontargeting control (NTC) siRNAs (Fig. 4A). ZIP1 knockdown led to around a 3-fold increase in RSV titers at 24 h p.i., whereas no difference in RSV titers was observed under the ZIP8 KD condition (Fig. 4B and C). The data suggest that downregulating ZIP1, which may impact zinc uptake from the extracellular medium, leads to better RSV infection, suggesting that zinc may act as an antiviral to perturb RSV infection.

### Zinc supplementation inhibits RSV replication.

Our results indicate that cellular zinc levels increase in RSV-infected cells, suggesting that either increased zinc levels benefit RSV infection or zinc induction is part of a host response to deal with RSV infection. We further investigated the relevance of cellular zinc in RSV infection by testing the effect of zinc supplementation and zinc depletion in A549 cells. We first determined zinc uptake in A549 cells by supplementing the cells with 25 μM to 100 μM zinc in serum-free medium (Fig. S4A). Next, we determined the efficiency of zinc uptake in serum-free media, 2% serum-containing media, and 10% serum-containing media. As expected, zinc uptake by cells, measured as increases in labile zinc levels, decreased with increasing concentrations of serum. ZP-1 signal intensity decreased from ∼2.5-fold to 1.5-fold with serum concentrations increasing from 0% to 2%. Zinc uptake, as measured by changes in the ZP-1 signal, was negligible in medium containing 10% serum under these conditions (Fig. S4B). We then tested whether zinc supplementation has any effect on cell viability by treating A549 cells with different concentrations of ZnSO_4_ from 25 μM up to 500 μM and assessing cell viability (Fig. S4C). Similarly, we also determined that 25 μM ZnSO_4_ had no effect on cell viability in SAECs (data not shown).

10.1128/mSphere.00447-20.4 FIG S4 Optimization of Zn treatment conditions. (A) A549 cells were treated with 25, 50, and 100 μM ZnSO_4_. Labile zinc levels were measured using ZP-1 at 24 h posttreatment. (B) A549 cells were treated with 100 μM ZnSO_4_ in serum-free medium and in 2% and 10% serum-containing media. Zinc uptake was measured at 24 h p.i. using zinpyr-1 staining by flow cytometry. (C) A549 cells were treated with different concentrations of ZnSO_4_, as indicated, in serum-free media. Cell viability was measured using the CellTiter-Glo luminescent cell viability assay. Data are from three independent experiments, and error bars indicate standard deviations from the means. **, *P* < 0.01; ***, *P* < 0.001. Download FIG S4, TIF file, 0.2 MB. Copyright © 2020 Khan et al. 2020 Khan et al. This content is distributed under the terms of the Creative Commons Attribution 4.0 International license.

**FIG 5:**
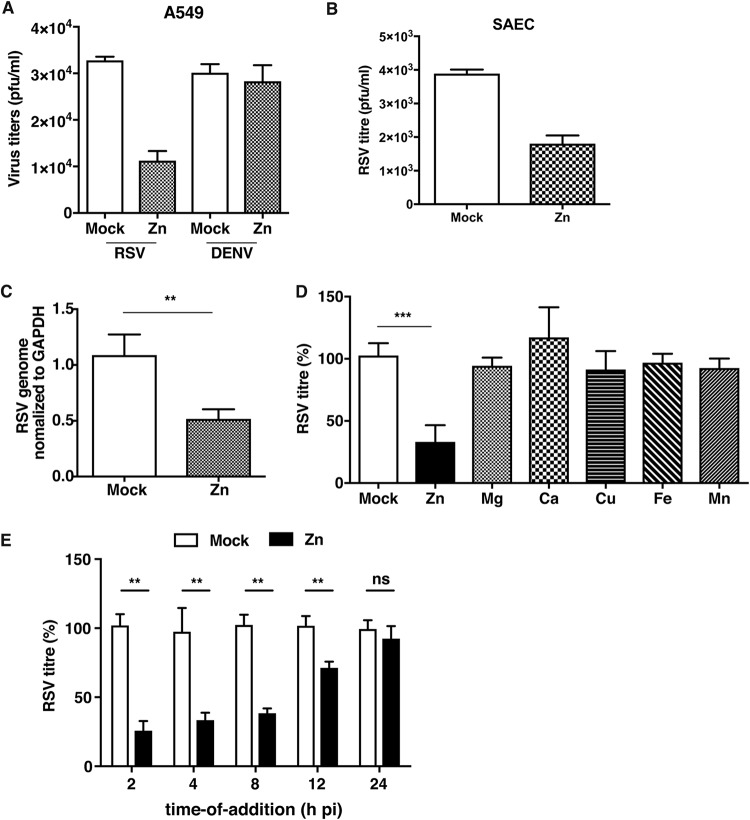
Zinc supplementation inhibits RSV infection. (A) A549 cells were infected with RSV and DENV at MOIs of 0.3 and 5, respectively, after adsorption of 100 μM added ZnSO_4_ . RSV and DENV titers were determined at 24 h p.i. (B) SAECs were infected with RSV at an MOI of 3, and 25 μM ZnSO_4_ was added after infection. RSV titers were determined at 24 h p.i. and are represented as numbers of PFU per milliliter. (C) RSV genome levels were determined under the zinc-supplemented condition at 24 h p.i. (D) A549 cells were infected with RSV at an MOI of 0.3. After adsorption, cells were treated with metal salts as indicated in the text. Viral titers were determined by plaque assay and are represented as percentages. (E) A549 cells were infected with RSV at an MOI of 0.3, and ZnSO_4_ was added at 2, 4, 8, 12, and 24 h p.i. Supernatants were collected at 24 h p.i. for the 2-, 4-, and 8-h conditions and at 48 h p.i. for the 12- and 24-h conditions. Viral titers are represented as percentages. Data are from at least two independent experiments. Error bars represent standard deviations of the means. **, *P* < 0.01; ***, *P* < 0.001; ns, not significant.

We next investigated the effect of zinc supplementation and zinc chelation on RSV infection. We infected A549 cells with RSV and DENV at MOIs of 0.3 and 5, respectively. After virus adsorption, cells were treated with 100 μM ZnSO_4_. Viral titers were measured at 24 h p.i. by plaque assay. We observed close to a 70% reduction in RSV titers under conditions of excess ZnSO_4_ in the medium, whereas DENV titers showed no significant change (Fig. 5A). Similar inhibitions of RSV titers were observed in primary SAECs infected with RSV and cultured in medium containing 25 μM ZnSO_4_ (Fig. 5B). To verify the effect of zinc on RSV genome replication, we treated A549 cells with ZnSO_4_ as described above and estimated RSV genome levels at 24 h p.i.; we observed a 50% reduction in viral RNA levels in cells treated with ZnSO_4_, suggesting that zinc inhibits RSV replication (Fig. 5C). We also tested whether this inhibition is specific to zinc or whether other divalent cations are also capable of inducing a similar inhibitory effect with RSV. RSV-infected A549 cells were cultured in the presence of 100 μM ZnSO_4_, magnesium chloride, calcium chloride, or copper chloride and 10 μM ferrous sulfate and manganese chloride. These concentrations were determined as noncytotoxic concentrations. No divalent cationic salt besides ZnSO_4_ showed any inhibitory effect on RSV infection (Fig. 5D). RSV inhibition by zinc was further confirmed by time-of-addition experiments wherein 100 μM ZnSO_4_ was added at 2, 4, 8, 12, and 24 h p.i. Cell supernatants were collected at 24 h p.i. for cells in which ZnSO_4_ was added at 2, 4, and 8 h p.i. In cells at which the times of addition were 12 h p.i. and 24 h p.i., supernatants were collected at 48 h p.i. The effect of addition of ZnSO_4_ on RSV titers gradually diminished with time. ZnSO_4_ added after 2 h p.i. and 4 h p.i. showed an ∼70% decrease in RSV titers, whereas addition of ZnSO_4_ at 8 h p.i. reduced RSV titers by 50%. No significant effect of ZnSO_4_ addition was observed beyond 12 h p.i. (Fig. 5E). These data suggest that addition of zinc affects early stages of the RSV life cycle.

### Zinc chelation enhances RSV infection.

We have recently reported that zinc depletion negatively affects DENV infection in Caco-2 cells (21). To address if zinc depletion affects RSV infection, we further standardized zinc depletion conditions in A549 cells using a cell-permeable zinc chelator, *N*,*N*,*N*′,*N*′-tetrakis(2-pyridinylmethyl)-1,2-ethanediamine (TPEN). We first determined the effect of TPEN treatment on cell viability by treating cells with TPEN at concentrations starting from 0.125 μM to 2 μM. We found that TPEN concentrations above 0.5 μM affected cell viability. We next measured the extent of reduction in labile zinc levels with TPEN treatment under these conditions. ZP-1 levels were down by 60% with 0.5 μM TPEN for 24 h compared to levels with a vehicle control (dimethyl sulfoxide [DMSO]) (Fig. S5A and S5B). These data suggest that regulation of zinc homeostasis is closely linked to cell viability and that both excess zinc and low zinc may affect cell fates.

10.1128/mSphere.00447-20.5 FIG S5 Standardization of zinc chelation by TPEN. (A) A549 cells were treated with 0.125 μM to 2 μM TPEN in serum-free medium. At 24 h posttreatment, cell cytotoxicity was measured using the CellTiter-Glo luminescent cell viability assay. (B) A549 cells were treated with 0.5 μM TPEN, and labile zinc levels were measured using ZP-1 staining. Data are from at least two independent experiments, and error bars indicates standard deviations from the means. **, *P* < 0.01. Download FIG S5, TIF file, 0.2 MB. Copyright © 2020 Khan et al. 2020 Khan et al. This content is distributed under the terms of the Creative Commons Attribution 4.0 International license.

**FIG 6:**
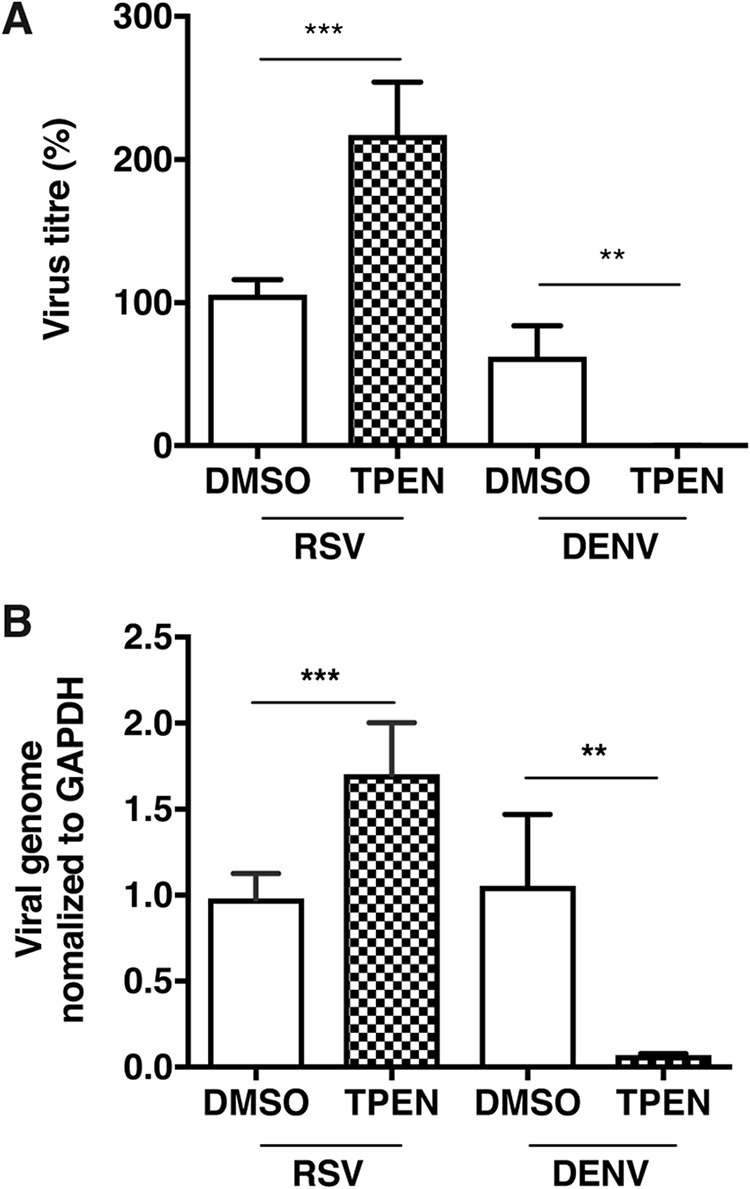
Zinc chelation enhances RSV infection. (A) A549 cells were infected with RSV and DENV at MOIs of 0.3 and 5, respectively. After adsorption, cells were treated with 0.5 μM TPEN. Viral titers from two independent experiments are represented as percentages. (B) RSV and DENV genome levels were measured using qRT-PCR. Data are from at least three independent experiments. Error bars represent standard deviations of the means. **, *P* < 0.01; ***, *P* < 0.001.

We infected A549 cells with RSV or DENV and then treated the cells with 0.5 μM TPEN. TPEN treatment led to a 2-fold increase in RSV titers but a nearly complete inhibition of DENV titers at 24 h p.i. (Fig. 6A). We quantitated RSV and DENV genome levels under these conditions by quantitative real-time PCR (qRT-PCR). As with virus titers, we observed an ∼2-fold increase in RSV RNA levels and a >90% reduction in DENV RNA levels at 24 h p.i. under zinc-depleted conditions (Fig. 6B). These data suggest that the cellular response to RSV infection results in an increase in intracellular zinc, which plays an antiviral role by inhibiting RSV replication.

### Zinc inhibits RSV as an antioxidant.

**FIG 7:**
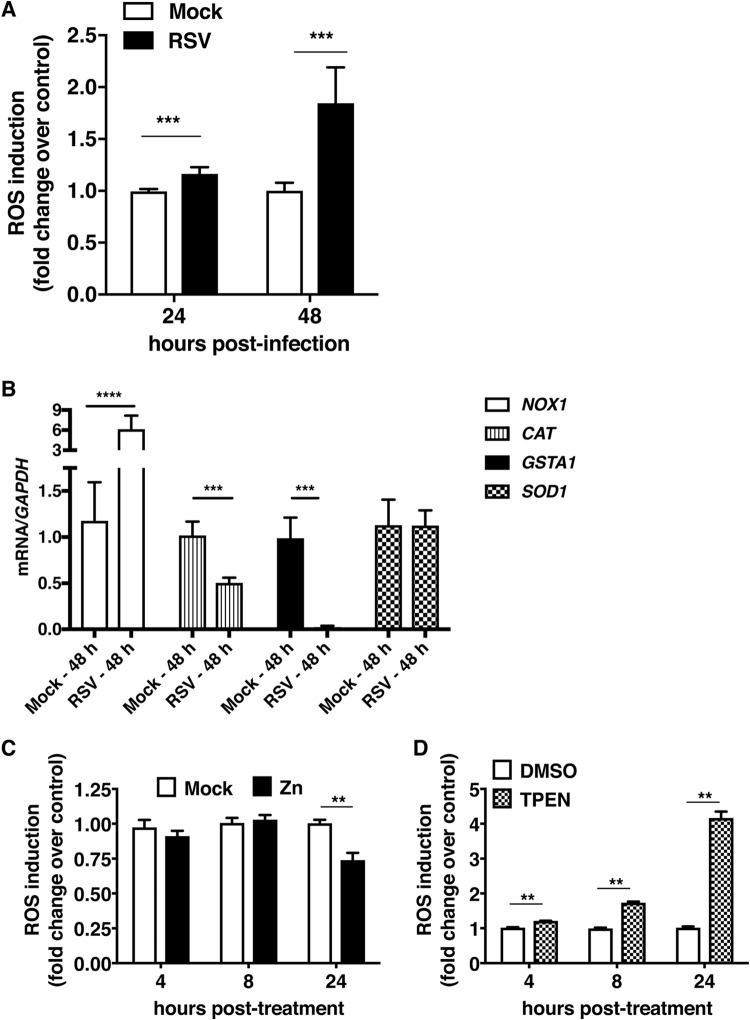
Interaction between zinc homeostasis, oxidative stress, and RSV infection. (A) A549 cells were infected with RSV at an MOI of 0.3. ROS levels were measured using the H_2_DCFDA method. (B) Gene expression were measured for *NOX1*, *CAT*, *GSTA2*, and *SOD1* at 48 h p.i. under the RSV infection condition. (C and D) A549 cells were treated with 100 μM ZnSO_4_ and 0.5 μM TPEN. ROS levels were measured at the indicated time points using H_2_DCFDA by flow cytometry. The fold changes in ROS levels are displayed for the zinc supplementation and zinc depletion conditions at the indicated time points. Data are from at least two independent experiments, and error bars indicate standard deviations from the means. **, *P* < 0.01; ***, *P* < 0.001; ****, *P* < 0.0001.

Respiratory viruses, such as RSV and influenza virus, induce oxidative stress in infected cells (22–24). Zinc has been shown to ameliorate oxidative stress, as some of the players in the antioxidant pathways, such as superoxide dismutase (SOD), require zinc for their functions (25). We hypothesized that induction of oxidative stress by RSV may be a trigger for increases in intracellular zinc levels and that zinc may exert anti-RSV activity as an antioxidant molecule. We first verified induction of oxidative stress by RSV in A549 cells. Oxidative stress was measured by estimating reactive oxygen species (ROS) in the cells by flow cytometry using H_2_DCFDA dye. We observed a modest 20% increase in ROS levels at 24 h p.i. However, by 48 h p.i., ROS levels increased by 2-fold in RSV-infected cells relative to levels after a mock infection (Fig. 7A). We next measured the expression levels of mRNAs of some of the antioxidant enzymes and found that RSV infection leads to induction of NADPH oxidase-1 (NOX1), which is a ubiquitously expressed enzyme involved in the synthesis of ROS. RSV infection also led to inhibition in mRNA levels of catalase (*CAT*) and glutathione *S*-transferase A2 (*GSTA2*) at later stages of infection which coincide with ROS induction. No effect was observed on superoxide dismutase-1 (*SOD1*) (Fig. 7B). These data suggest that induction of *NOX1* and suppression of antioxidant enzymes during RSV infection leads to upregulation of ROS.

**FIG 8:**
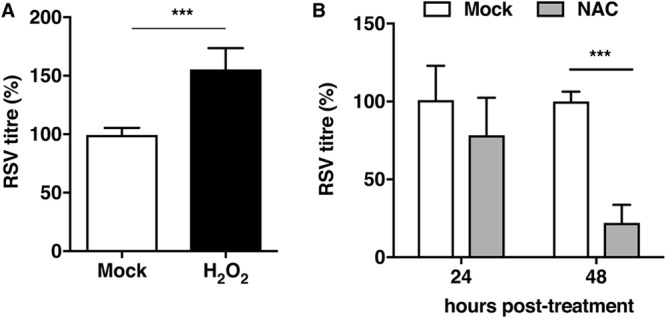
Oxidative stress modulates RSV infection. (A and B) A549 cells were infected with RSV at an MOI of 0.3. H_2_O_2_ (300 μM) and NAC (10 mM) were added. RSV titers were measured at 24 h p.i. for the H_2_O_2_ treatment condition, whereas 24-h p.i. and 48-h p.i. cell supernatants from the NAC treatment were subjected to plaque assay. Viral titers are represented as percentages. Data are from at least two independent experiments, and error bars indicate standard deviations from the means. ***, *P* < 0.001.

The kinetics of induction of ROS in RSV-infected cells correlated with increases in labile zinc levels observed in these cells. As zinc has been shown to act as an antioxidant (26), we speculated that the increase in labile zinc levels may be part of a host response to counter the damage by RSV-induced oxidative stress. To test this, we first determined whether modulation of zinc levels by zinc supplementation or chelation could affect basal ROS levels. A549 cells were treated with 100 μM ZnSO_4_ and 0.5 μM TPEN. Intracellular ROS levels were measured at 4 h, 8 h, and 24 h posttreatment using H_2_DCFDA. Although excess zinc in the culture medium had no effect on basal ROS levels in the first 8 h of treatment, the continued presence of zinc for 24 h lowered basal ROS levels by 25% (Fig. 7C). In contrast to this, zinc chelation by TPEN led to significant increases in ROS levels in a time-dependent manner, and we observed a 4-fold increase in ROS levels by 24 h of TPEN treatment (Fig. 7D). To further confirm the role of oxidative stress in RSV infection, cells infected with RSV were treated with 300 μM H_2_O_2_ (one of the ROS) and virus titers were measured at 24 h p.i. As observed in the case of zinc chelation, addition of H_2_O_2_ led to a modest but significant increase in RSV titers at 24 h p.i. (Fig. 8A). We further determined the importance of oxidative stress by adding 10 mM *N*-acetyl cysteine (NAC), which is a potent antioxidant compound, after virus adsorption and RSV titers in the supernatant were measured at 24 and 48 h p.i. by plaque assay. Unlike with the addition of ROS, the presence of NAC during infection suppressed RSV infection (Fig. 8B). These results indicate that the induction of ROS during RSV infection is beneficial for virus replication and that the cells respond to counter this effect by modulating zinc homeostasis and increase the cellular uptake of zinc, which acts to suppress virus-induced oxidative stress, thereby limiting virus replication.

## DISCUSSION

Zinc homeostasis is regulated by zinc influx and efflux transporters, which modulate cellular zinc levels in response to various stimuli, such as inflammation, stress responses, and growth factors, and changes in intracellular labile zinc levels constitute an integral part of this modulation (27–29). Free zinc is proposed to act as a second messenger regulating signal transduction pathways, further underscoring the importance of zinc homeostasis in health and disease (30, 31). We demonstrate that RSV infection leads to increases in labile zinc levels in A549 cells, primary small airway epithelial cells, and epithelial cells isolated from nasopharyngeal aspirates (NPA) of children with RSV infection. In contrast to this, DENV infection had no effect on zinc homeostasis in A549 cells, suggesting that, in lung epithelial cells, modulation of zinc homeostasis is specific to RSV. We demonstrate that the total zinc content in RSV-infected cells is increased, suggesting an increase in zinc uptake by *ZIP* family transporters. We show that knockdown of *ZIP1* but not *ZIP8* led to increases in RSV titers in the supernatants, suggesting that zinc uptake during RSV infection may act as an antiviral response. There are 14 *ZIP* transporters in mammalian cells, and we have not explored the role of other *ZIP* family members in our study. We further prove the antiviral effect of zinc ions by zinc supplementation and zinc chelation studies and show that these treatments produce opposing results with RSV titers. Supplementation of culture media with excess zinc, but not with any other divalent cation, inhibited RSV infection, while zinc chelation by TPEN led to increased virus titers. Increases in labile zinc levels in RSV infection coincided with an induction of oxidative stress, suggesting a critical role for zinc homeostasis in the host response to RSV infection. We also show that RSV infection actively induces oxidative stress by upregulation of *NOX1*, which is involved in the generation of ROS and also in the downregulation of *CAT* and *GSTA2*, which are components of antioxidant pathways. Furthermore, we show that oxidative stress is a proviral pathway in the case of RSV infection, as induction of ROS by zinc chelation or addition of ROS (H_2_O_2_) exogenously led to increases in virus titers, whereas antioxidants such NAC inhibited RSV.

Zinc is a stable divalent cation and does not directly undergo redox reactions but is involved in regulating the oxidant/antioxidant balance (32). The cytosolic pool of zinc is regulated by metallothioneins, and the redox status of the cells plays an important role in maintaining this labile zinc pool. Therefore, induction of oxidative stress by RSV may also act as a trigger for metallothioneins to release zinc into the cytoplasm to maintain the cellular redox state (26, 33). Increases in labile zinc levels appear to be an antiviral response, as zinc supplementation led to inhibition of RSV infection, whereas mimicking zinc deficiency by TPEN or *ZIP1* knockdown proved to be beneficial for RSV infection. Increased oxidative stress is one of the clinical manifestations of zinc deficiency, which can be corrected by zinc supplementation (34, 35). Oxidative stress may influence viral infections in more than one way depending on the host pathways and compartments that are usurped by the viruses for genome replication, translation, assembly, and egress (36, 37). In Saccharomyces cerevisiae, induction of oxidative stress by hydrogen peroxide led to increases in the expression of a number of proteins involved in maintaining the redox homeostasis, such as manganese superoxide dismutase (MnSOD), Cu/Zn-SOD, catalase, components of proteasome, and heat shock proteins. It was also observed that under oxidative-stress conditions, carbohydrate metabolism was redirected from glycolysis to generation of NADPH to deal with the redox imbalance. Therefore, viruses such as DENV, which depend on glycolysis as a source of energy during replication (38), may avoid induction of ROS, while RSV actively downregulates antioxidant pathways (39). Interestingly, treatment of mice or lung epithelial cells with Toll-like receptor (TLR) agonists led to induction of ROS, which inhibited influenza virus replication, clearly demonstrating the contrasting roles of ROS in respiratory virus infections (40). Based on our results, we propose that RSV infection induces oxidative stress, which creates a favorable environment for RSV replication and spread. Cells counter this response by enhancing cellular zinc levels by increased uptake by zinc transporters (*ZIP1* and possibly other *ZIP*s). Close to 2 billion people worldwide consume zinc-deficient diets, and 1 to 4% of the deaths, mostly in infants, occur in zinc-deficient subjects due to compromised immune systems. We speculate that children with zinc deficiency may be more susceptible to infections that induce and thrive under oxidative stress, such as RSV, and therefore, zinc supplementation may have a stronger impact in such infections due to its antioxidant function. In addition, whether transient treatment with antioxidants may also provide the same beneficial effect as zinc in RSV infections remains to be explored.

## MATERIALS AND METHODS

### Ethics statement.

The study protocol was approved by the institutional ethics committees (human research) of both the participating institutes [Translational Health Science and Technology Institute protocol THS 1.8.1/(32), dated 5 February 2015; All India Institute of Medical Sciences (AIIMS) protocol IEC/NP-352/08.10.2014,RP-16/2014, dated 26 November 2014). Nasopharyngeal aspirates (NPA) from RSV-infected and control samples were collected at the Department of Pediatrics, AIIMS, New Delhi, India. Written informed consents were obtained from the parents or guardians of the children. NPA samples were screened for RSV infection using a strip-based kit (Coris BioConcept) and further confirmed by qRT-PCR. Briefly, RNA was isolated from NPA samples using a QIAamp MinElute virus spin kit (Qiagen), and RT-PCR was set up using a Flu-RSV kit (Fast-Track Diagnostics).

### Cells and viruses.

BHK and HEp-2 cells were procured from the American Type Culture Collection (ATCC) and cultured as described previously, and virus strains used in the study were described previously (41). A549 cells were procured from the European Collection of Authenticated Cell Cultures (ECACC) and cultured in Dulbecco’s modified Eagle medium (DMEM) with 10% fetal bovine serum (FBS), penicillin, streptomycin, glutamine, and nonessential amino acids. Respiratory syncytial virus (Long strain) was procured from the ATCC (ATCC VR-26). RSV titers were estimated by plaque assays on HEp-2 cells by following the same procedure as for DENV (41). For UV inactivation, RSV stock was diluted 1:100 in 1 ml of MEM and divided in two 3-cm dishes. One dish was put inside the UV chamber and kept for 10 min × 3 times on ice at 9,999 × 10^2^ μJ/cm^2^ with 3-min intervals between each cycle. The other dish was kept on ice as a control in a biosafety cabinet. After the inactivation process, virus was aliquoted in small volumes and stored at −80°C. UV-irradiated virus showed no plaques in plaque assay. All cell lines were checked routinely for mycoplasma contamination using an RT-PCR-based method (42). The Madin-Darby canine kidney (MDCK)-London cells (IRR-FR-58) and influenza A virus (IAV) strain A/Mexico/4108/2009 (H1N1) pdm09 (IRR-FR-245) used in the study were obtained from the International Reagent Resource. MDCK-London cells were grown in advanced MEM (Gibco) with 10% FBS along with penicillin-streptomycin and l-glutamine (Gibco). For IAV infection, 10% FBS was replaced with 3% bovine serum albumin (BSA), and 1 M HEPES, pH 7.4 (0.025%), sodium bicarbonate (0.15%), and DEAE dextran (5 μg/ml) were added. Virus stock generation and hemagglutination units (HAU) were estimated as described previously (43, 44). Briefly, MDCK-London cells were infected at 37°C for 1 h to allow for viral internalization, followed by the addition of infection medium with 0.5 μg/ml *N*-tosyl-l-phenylalanine chloromethyl ketone (TPCK)-treated trypsin (Sigma, USA). IAV titers from supernatants were estimated by plaque assays on MDCK cells by the same protocol used for DENV and RSV. For labile zinc measurement experiments, A549 cells were infected with IAV at MOIs of 0.5 and 1 for 1 h at 37°C to allow for viral internalization, followed by the addition of infection medium with 0.5 μg/ml TPCK-treated trypsin. Virus titers from supernatants were collected at 24 h p.i. and 48 h p.i., and HAU were determined as described previously (43, 44).

### Treatment of cells.

Cells were treated with 100 μM ZnSO_4_ or 0.5 μM *N*,*N*,*N*′,*N*′-tetrakis(2-pyridinylmethyl)-1,2-ethanediamine (TPEN) (Sigma) in serum-free media for the periods indicated in the figures. Labile zinc levels were measured by flow cytometry as described in Text S1 in the supplemental material. To study the effect of zinc and TPEN on virus infection, 100 μM ZnSO_4_ or 0.5 μM TPEN was added in serum-free medium after infection. At time points indicated in the figures, the supernatant was collected for estimating virus titer by plaque assay and cells were collected for quantitative real-time PCR (qRT-PCR) as described previously (21). For H_2_O_2_ and *N*-acetyl cysteine (NAC) treatment, infected cells were incubated with 300 μM H_2_O_2_ (Merck) in serum-free media or with 10 mM NAC for the periods indicated in the figures, and the effect on virus titers was assessed by plaque assay.

10.1128/mSphere.00447-20.6 TEXT S1 Supplemental materials and methods for the cytotoxicity assay and immunofluorescence. Download Text S1, DOCX file, 0.02 MB. Copyright © 2020 Khan et al. 2020 Khan et al. This content is distributed under the terms of the Creative Commons Attribution 4.0 International license.

### Quantitative real-time PCR.

Cells were harvested for isolation of total RNA as described previously (41). Briefly, RNA was isolated using RNAiso Plus (TaKaRa) per the manufacturer’s instructions and reverse transcribed using random hexamers. The reverse transcription product was further amplified using NOX1, catalase, GSTA1, and SOD1 using a PowerUp SYBR kit (Applied Biosystems). Human GAPDH (glyceraldehyde-3-phosphate dehydrogenase) primer mix was used as the housekeeping control. Data were analyzed using the ΔΔ*C_T_* method, where *C_T_* is threshold cycle. The list of primers used in the study is provided in Table S1. For RSV genome quantitation, cDNA was made using the reverse primer with a genomic DNA (gDNA) eraser kit (TaKaRa), followed by qRT-PCR using the Premix *Ex Taq* master mix (TaKaRa).

10.1128/mSphere.00447-20.7 TABLE S1 List of primers used in the study. Download Table S1, DOCX file, 0.01 MB. Copyright © 2020 Khan et al. 2020 Khan et al. This content is distributed under the terms of the Creative Commons Attribution 4.0 International license.

### siRNA knockdown.

siRNA transfections were carried out as described previously (45). Briefly, 5 nM concentrations of ZIP1 and ZIP8 siRNA (Dharmacon) were mixed with Opti-MEM (Life Technologies) and 1 μl of Lipofectamine RNAiMax to a total volume of 100 μl in a 24-well plate. Forty thousand cells were transfected in suspension in triplicates and seeded into 24-well plates. Knockdown efficiency was monitored by RT-PCR at 48 h posttransfection. At forty-eight hours posttransfection, cells were infected with RSV at an MOI of 0.3, and at 24 h p.i., viral titers in the supernatants were measured by plaque assays.

### Flow cytometry.

**(i) Labile zinc level measurement.** Labile Zn levels in the cells were estimated as described before (21). Briefly, cells were washed once with phosphate-buffered saline (PBS) after either treatment or infection for the periods indicated in the figures, following which they were detached using trypsin (80 to 120 μl) (Gibco) and collected after the addition of the defined trypsin inhibitor (Gibco). Cells were resuspended in staining medium (DMEM without phenol red supplemented and with 2 mM l-glutamine; Gibco). Cells were stained using either 5 μM Fluozin-3-AM (Molecular Probes) or 2.5 μM ZinPyr-1 (ZP-1) (Santa Cruz) in the staining medium. For ZP-1 staining, medium containing 1 mM EDTA was added to chelate any extracellular zinc during staining. Cells were incubated for 30 min at 37°C in a CO_2_ incubator and mixed every 10 min. After 30 min, fixable viability stain eFluor780 (Becton, Dickinson Biosciences) was added to the cells at a 1:500 dilution, prepared in the staining medium, and incubated for a further 10 min at 37°C in the CO_2_ incubator. Cells were washed using fluorescence-activated cell sorter (FACS) buffer (PBS containing 0.25% FBS), and samples were acquired in a FACS Canto II apparatus (Becton, Dickinson). The amounts of labile zinc present in the cells are presented as the mean fluorescence intensities of FLZ-3 and ZP-1.

**(ii) Annexin-V staining.** Apoptosis was induced by treating A549 cells at 56^○^C for 15 min. Cells were treated with Accutase for 3 min and collected. Cells were then centrifuged at 800 × *g* for 5 min. Cells were then stained with 2.5 μl of Annexin-V–fluorescein isothiocyanate (FITC; eBioscience) in 1× Annexin-V binding buffer for 15 min at room temperature according to the manufacturer’s protocol. Cells were then stained with eFluor-780 fixable viability dye for 15 min, washed with FACS buffer (PBS containing 0.25% FBS), and acquired in the FACS Canto II system.

**(iii) ROS measurement.** Cells were cultured in 48-well plates and treated with 100 μM ZnSO_4_ or 0.5 μM TPEN posttreatment for the times indicated in the figures. Cells were washed twice with PBS and detached by trypsinization. Cells were then incubated with medium containing 1% FBS and a 10 μM concentration of the carboxyl analog of 2′,7′-dichlorodihydrofluorescein diacetate (carboxy-H_2_DCFDA; Molecular Probes) for 1 h at 37°C. Cells were washed with FACS buffer, and ROS levels were measured using the FACS Canto II system.

### ICP-MS.

A549 cells were grown in a 12-well plate. Cells were infected with RSV at an MOI of 0.3. At 48 h p.i., cells were washed with PBS. Later cells were collected in 1 ml of 0.1% SDS solution. The lysates were then filtered with a 0.4-μm filter. Multielement standards (Merck) were used from 500 ppb to 1.95 ppb using the 2-fold dilution method in deionized water. Acquisition was carried out using the X series 2 inductively coupled plasma mass spectrometer (ICP-MS; Thermo Fisher Scientific). Protein estimation was carried out using a bicinchoninic acid (BCA) kit (Pierce). Data were normalized to total protein content and are represented as parts per billion per milligram of protein.

### Data analysis.

Data were analyzed and graphs were prepared using Prism 7 software (GraphPad Software Inc.). All experiments were performed in two or more replicates, and graphs represent results from at least two independent experiments performed with two or more replicates each time; values are presented as means ± SDs. Statistical significance was estimated by *t* tests (unpaired, nonparametric), two-way analysis of variance (ANOVA), or the Mann-Whitney test.
