# Supplementary figures and images for: Respiratory Syncytial Virus-Induced Oxidative Stress Leads to an Increase in Labile Zinc Pools in Lung Epithelial Cells
Source: mSphere. 2020 May 27;5(3):e00447-20. doi: 10.1128/mSphere.00447-20 (PMC7253603; doi:10.1128/mSphere.00447-20)

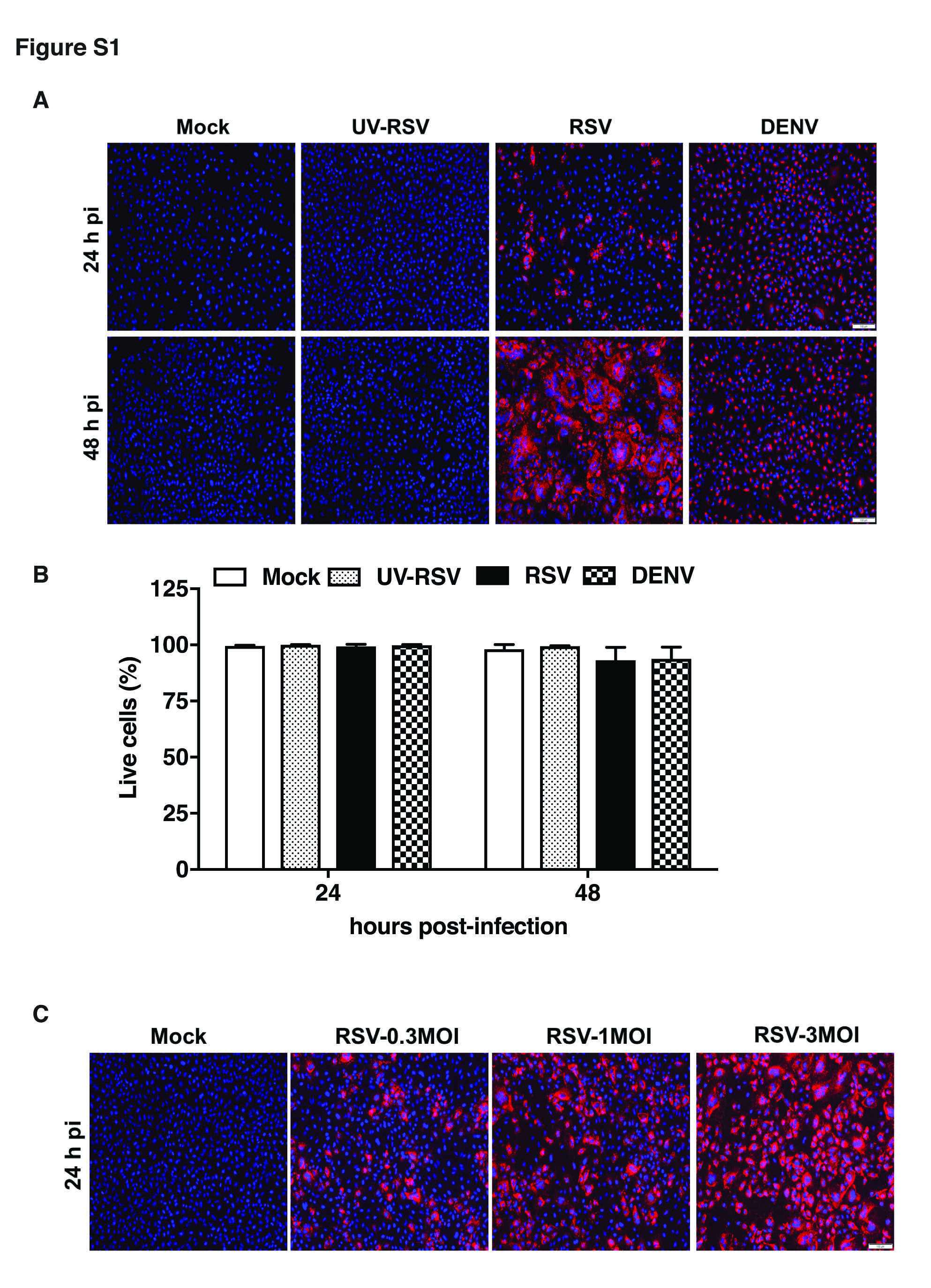

Supplement: FIG S1 [file mSphere.00447-20-sf001.jpg]

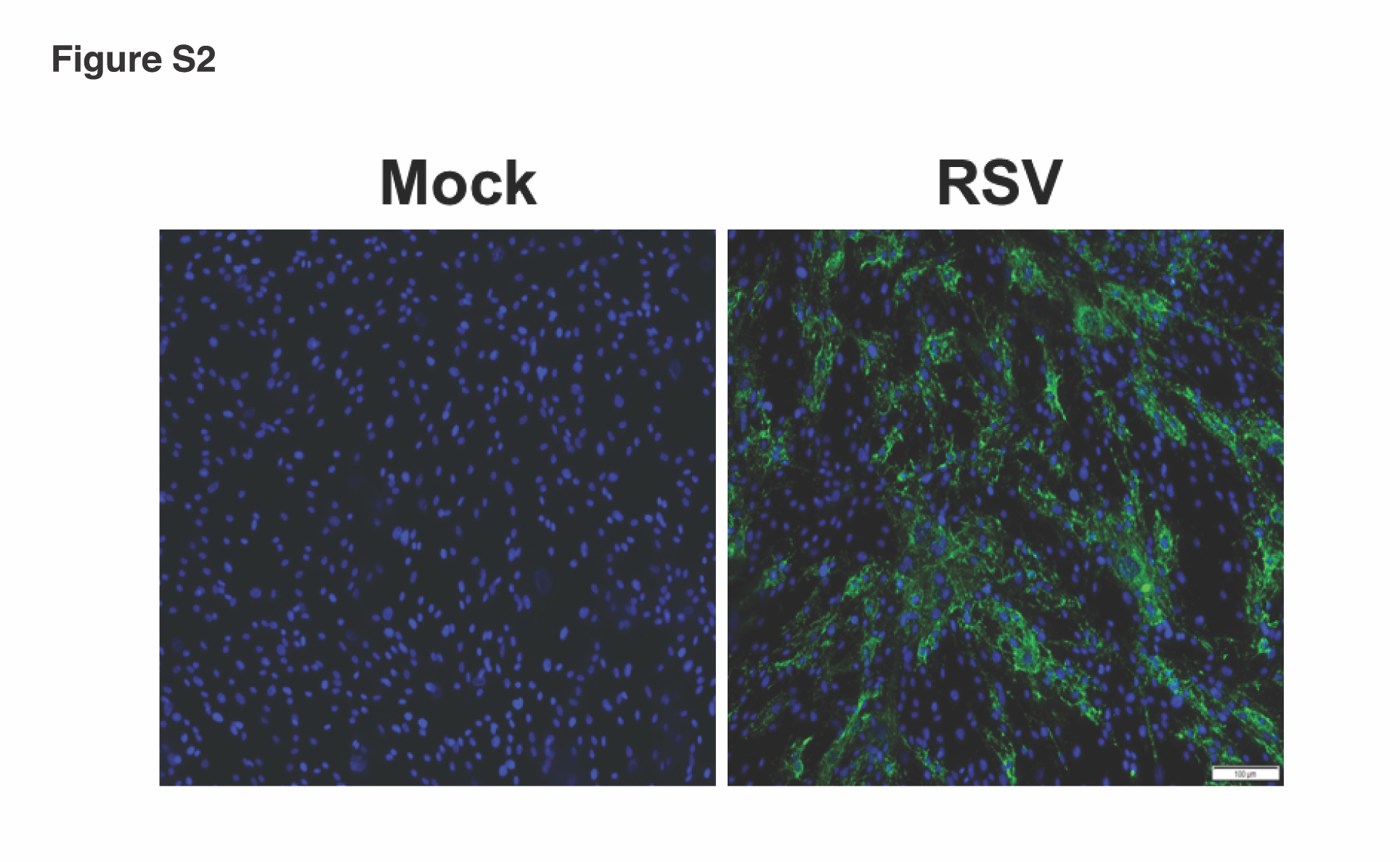

Supplement: FIG S2 [file mSphere.00447-20-sf002.tif]

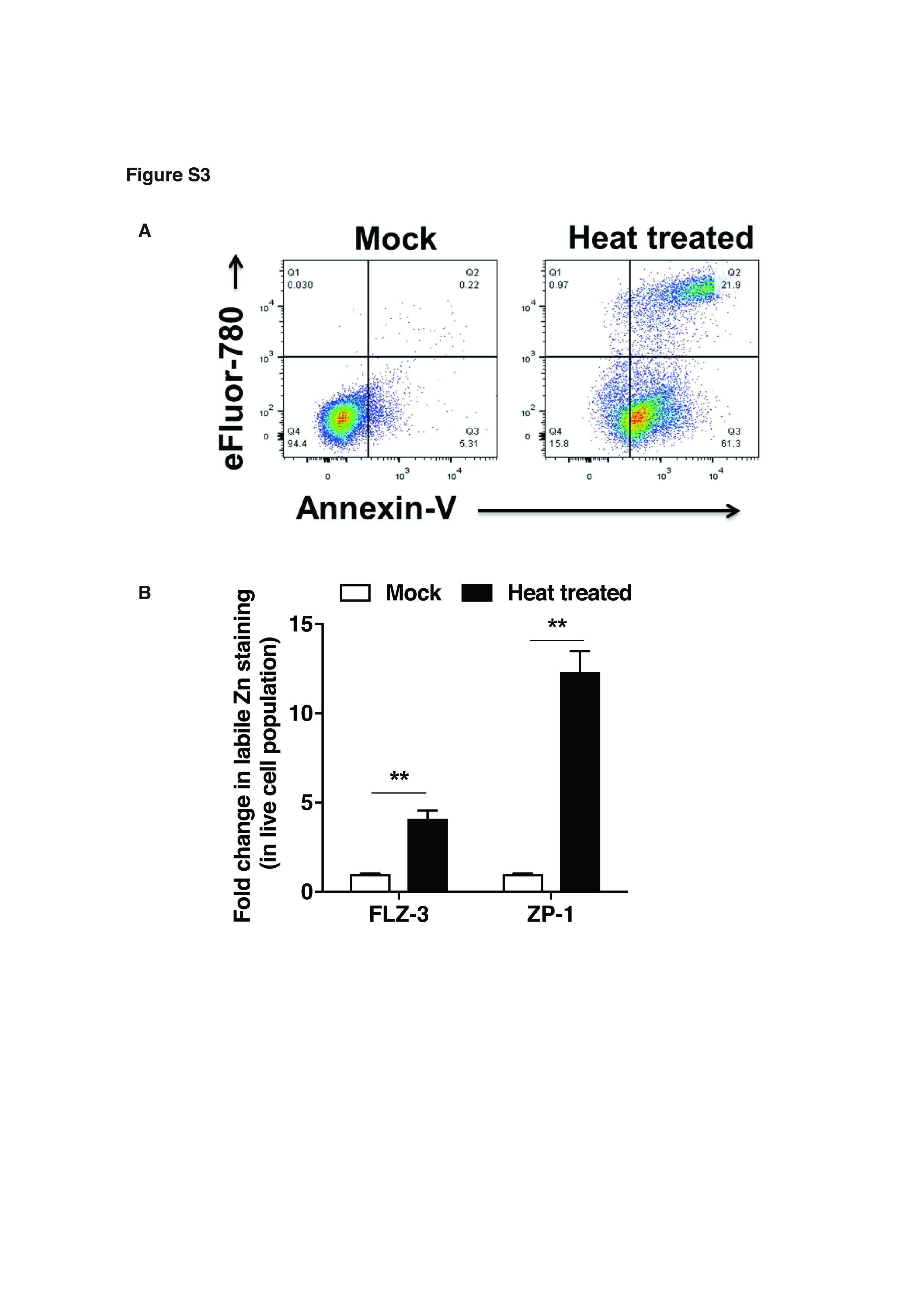

Supplement: FIG S3 [file mSphere.00447-20-sf003.tif]

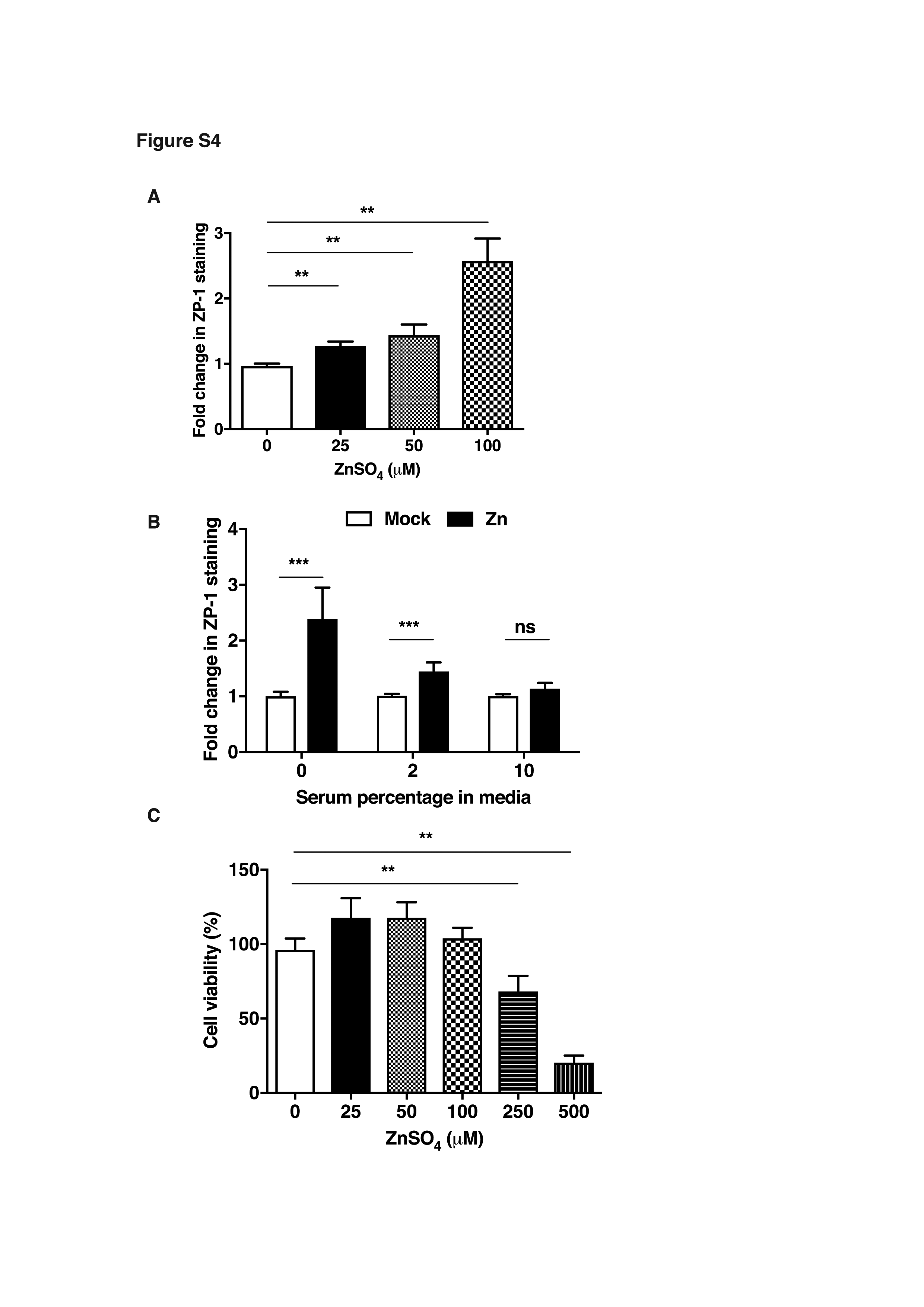

Supplement: FIG S4 [file mSphere.00447-20-sf004.tif]

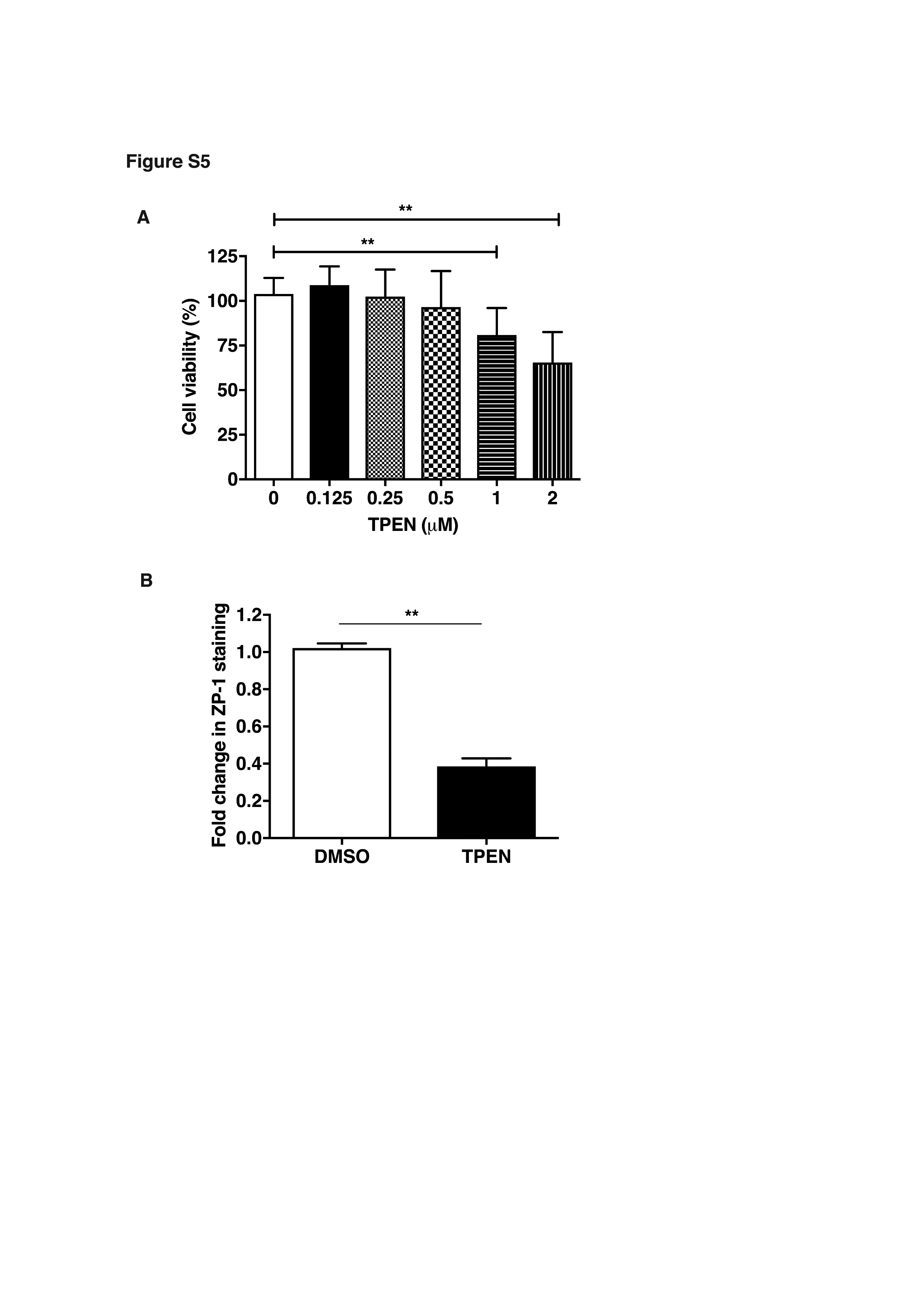

Supplement: FIG S5 [file mSphere.00447-20-sf005.tif]
