# Supplementary material for: Respiratory Syncytial Virus-Induced Oxidative Stress Leads to an Increase in Labile Zinc Pools in Lung Epithelial Cells
Source: mSphere. 2020 May 27;5(3):e00447-20. doi: 10.1128/mSphere.00447-20 (PMC7253603; doi:10.1128/mSphere.00447-20)
Supplement: TEXT S1 [file mSphere.00447-20-s0001.docx]

**SUPPLEMENTARY INFORMATION**

**MATERIALS AND METHODS**

**Cytotoxicity assay**

# A549 cells were plated in 96-well plate. Cells were treated with different concentration of ZnSO_4_ and TPEN for 24 h. Cytotoxicity was determined using CellTiter-Glo® Luminescent Cell Viability Assay (Promega) according to the manufacturers’ protocol.

**Immunofluorescence**

A549 cells were infected with DENV and RSV at indicated MOI and respected time points. Cells were fixed in methanol at -20^○^C. Cells were then stained with pan-flavivirus primary antibody (4G2) for DENV and RSV-F antibody (MCA490-AbdSerotech) for RSV infection followed by anti-mouse 568 secondary antibody. Cell nuclei were stained with DAPI. Images were acquired using inverted fluorescence microscope at 10X magnification (Olympus IX83).
