# Supplementary material for: Respiratory Syncytial Virus-Induced Oxidative Stress Leads to an Increase in Labile Zinc Pools in Lung Epithelial Cells
Source: mSphere. 2020 May 27;5(3):e00447-20. doi: 10.1128/mSphere.00447-20 (PMC7253603; doi:10.1128/mSphere.00447-20)
Supplement: TABLE S1 [file mSphere.00447-20-st001.docx]

Supplementary Table S1: List of RT-PCR primers used in the study

| **Gene** | **Primer sequence (5’ to 3’)** |
| --- | --- |
| *GAPDH* | FP: CCACTCCTCCACCTTTGAC  RP: ACCCTGTTGCTGTAGCCA |
| *NOX1* | FP: CTGTTGCCTAGAAGGGCTCC  RP: ACAGGCCAATGTTGACCCAA |
| *CAT* | FP: TCTCACCAAGGTTTGGCCTC  RP: CGGTGAGTGTCAGGATAGGC |
| *GSTA2* | FP: TTCTGCCCCGTATATTGG  RP: AGCCACAAAGGTGACAGC |
| *SOD1* | FP: CGCACACTGGTGGTCCAT  RP: TGGGCGATCCCAATTACACC |
| *ZIP1* | FP: TGAGCCTAGTAAGCTGTTTCGC  RP: CAGGGCCTCATCTATGGCA |
| *ZIP8* | FP: AATGATTGACAAAGCCCAAC  RP: TTCATGGTTTCAAAAGGGTG |
| RSV-NS1 | FP: TGCTTTGGCTAAGGCAGTGA  RP: TGTAGCACTGGCATTGTTGTG  Probe-FAM: CATTGTGTTTGTGCATGTTATTAC-TAMRA |
